# When the Skin Tells a Pulmonary Story: A Case of Neurofibromatosis Type 1-Associated Pulmonary Hypertension

**DOI:** 10.7759/cureus.98338

**Published:** 2025-12-02

**Authors:** Yasser Hegazy, Alshaimaa Abdallah, Ahmed Salem, Ali Assaker, Ahmed Elmogy

**Affiliations:** 1 Internal Medicine, Icahn School of Medicine at Mount Sinai, Queens Hospital Center, New York, USA; 2 Radiology, Cairo University, Cairo, EGY; 3 Internal Medicine, CarePoint Health Bayonne Medical Center, Bayonne, USA

**Keywords:** multifactorial pulmonary hypertension, neurofibromatosis 1, neurofibromatosis associated diffuse lung disease, pulmonary hypertension, type 5 pulmonary hypertension

## Abstract

Neurofibromatosis type 1 (NF1) is a multisystem autosomal dominant disorder with cutaneous, neurologic, and skeletal manifestations. Pulmonary hypertension (PH) is an uncommon but severe complication of NF1 and is classified as Group 5 PH due to its multifactorial and incompletely understood pathogenesis. We report a case of a 24-year-old female with a known history of NF1 who presented with progressive dyspnea, orthopnea, and lower extremity edema. Physical examination revealed classical NF1 features, including multiple café-au-lait macules, cutaneous neurofibromas, axillary freckling, and skeletal deformity. Imaging studies demonstrated severe pulmonary hypertension with enlarged pulmonary arteries, right ventricular dilation, and extensive cystic changes in both lungs, in the absence of thromboembolic disease. Echocardiography confirmed elevated right-sided pressures and right heart strain. Based on clinical, radiologic, and echocardiographic findings, a diagnosis of Group 5 PH associated with NF1 (PH-NF1) was made. Targeted PH therapy (endothelin receptor antagonists and phosphodiesterase-5 (PDE-5) inhibitors) was considered; however, due to limited evidence of benefit and potential risk in NF1-associated parenchymal lung disease, therapy was deferred in favor of conservative management with oxygen therapy and diuretics, and the patient was referred for pulmonary and cardiac rehabilitation. This case highlights the diagnostic complexity and therapeutic challenges associated with PH-NF1, a condition likely driven by a combination of vascular remodeling, parenchymal destruction, and genetic factors. Early recognition and multidisciplinary management are essential to improving outcomes in patients with this rare and progressive complication of NF1.

## Introduction

Neurofibromatosis type 1 (NF1) is a common autosomal dominant genetic disorder, with an estimated prevalence of one in 3,500 individuals. It exhibits complete penetrance and is characterized by a broad spectrum of clinical manifestations, including cutaneous findings such as café-au-lait macules and neurofibromas, optic pathway gliomas and other central nervous system tumors, skeletal dysplasia, cognitive impairments, and an elevated risk for certain malignancies beyond the nervous system [1]. Although respiratory involvement in NF1 is uncommon, reported pulmonary manifestations include intrathoracic neurofibromas, infiltrative lesions, cystic changes, bullae, and emphysematous alterations. Pulmonary hypertension (PH) is a rare but recognized complication, with only scattered case series and an estimated prevalence of approximately 1% or less among affected individuals. NF1-associated PH (PH-NF1) is classified under WHO Group 5, a category reserved for conditions with multifactorial or poorly understood mechanisms, because PH in NF1 may result from a combination of primary vasculopathy, including plexiform and proliferative arteriolar lesions, and secondary effects of parenchymal lung destruction that diminish the functional pulmonary vascular bed [2]. Notably, there is a lack of evidence-based guidance on the use of pulmonary arterial hypertension (PAH)-specific therapies in this population, and current management strategies focus primarily on treating the underlying disease [3]. In this report, we present a rare case of PH in a young female patient with NF1, a particularly unusual presentation given that PH-NF1 typically manifests later in life, contributing to the limited literature and highlighting the diagnostic challenges of this rare condition.

## Case presentation

A 24-year-old female presented with worsening shortness of breath of one month's duration. She reported intermittent exertional dyspnea for several years, beginning after an episode of spontaneous tension pneumothorax at age 17, which was treated with chest tube drainage. No follow-up imaging or pulmonary evaluation occurred at that time. Over the month preceding admission, she reports dyspnea on minimal exertion and orthopnea, but no paroxysmal nocturnal dyspnea. She also reported bilateral, painless, pitting edema of both lower limbs in the absence of a recent history of surgery or immobilization. She complains of right hypochondrial pain worsening on exertion. In addition, she reported having pigmented skin lesions of five-year duration and multiple variable-sized skin swellings for which she did not seek any previous consultation. Family history was significant for recurrent seizures in her sibling, which was not investigated regarding an underlying diagnosis.

**Figure 1:**
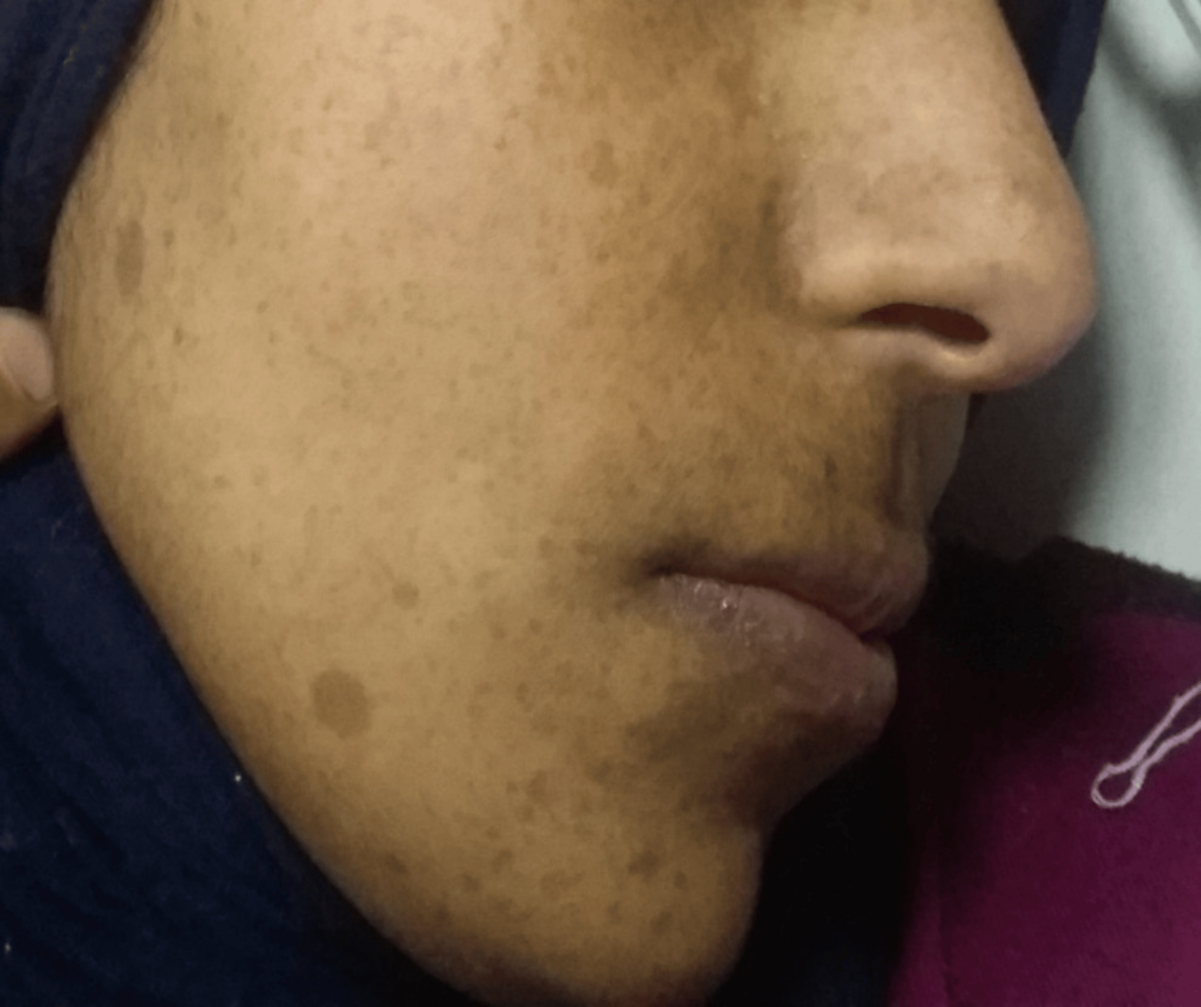
Multiple areas of facial skin pigmentation “café au lait” patches

**Figure 2:**
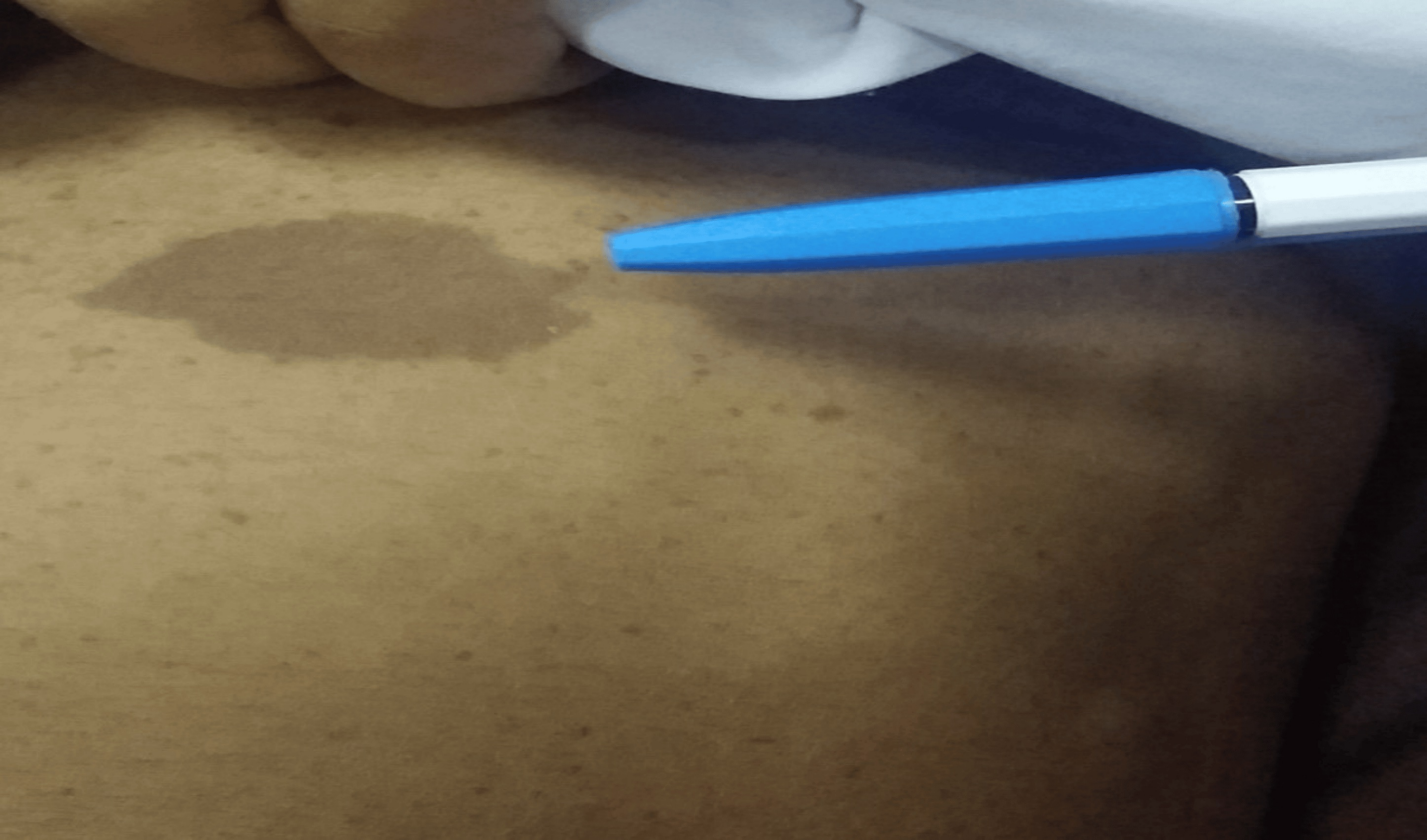
Multiple abdominal skin café au lait spots with a large macule

**Figure 3:**
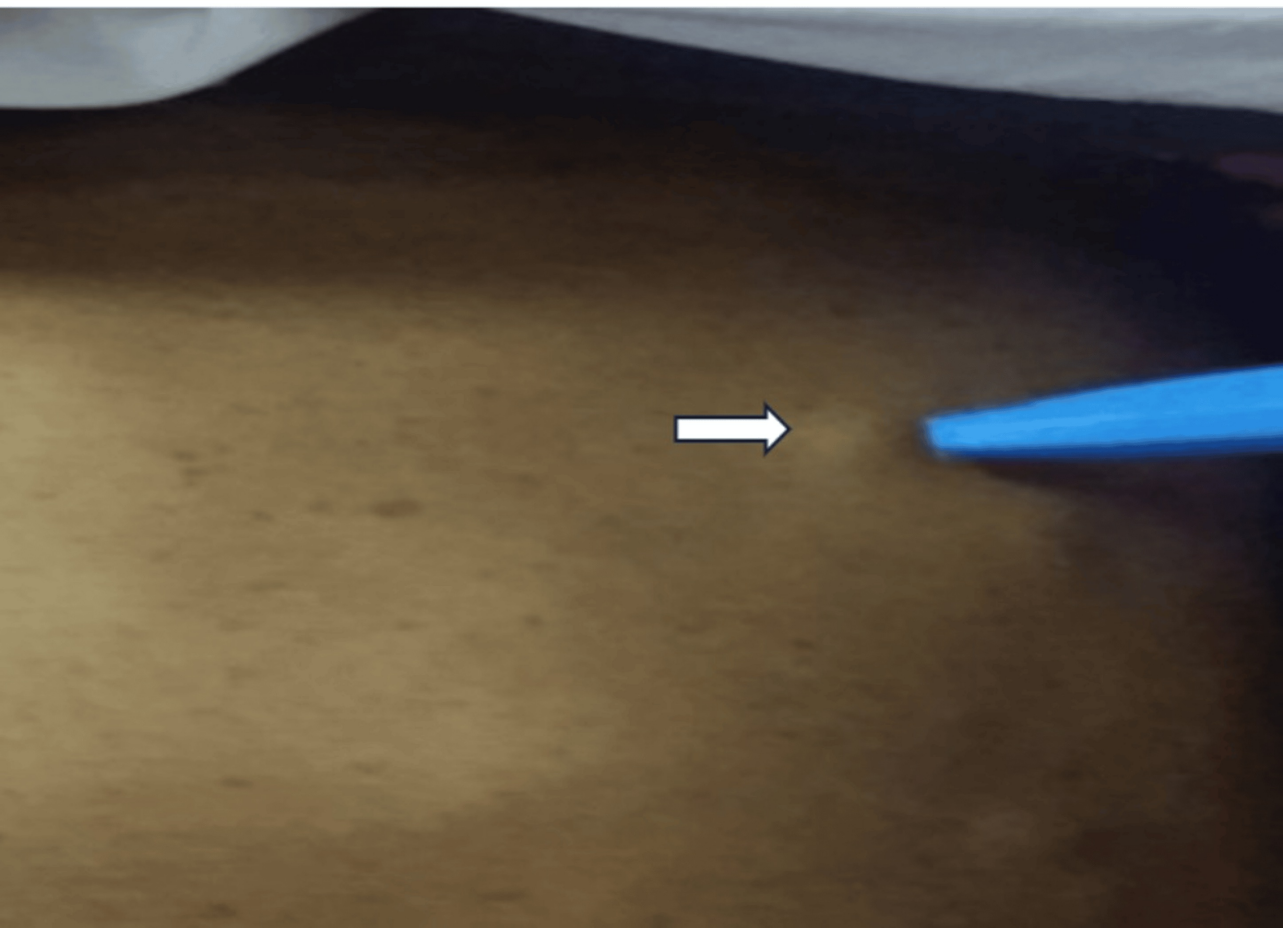
One of the multiple variable-sized subcutaneous swellings, “neurofibromas” (arrow)

**Figure 4:**
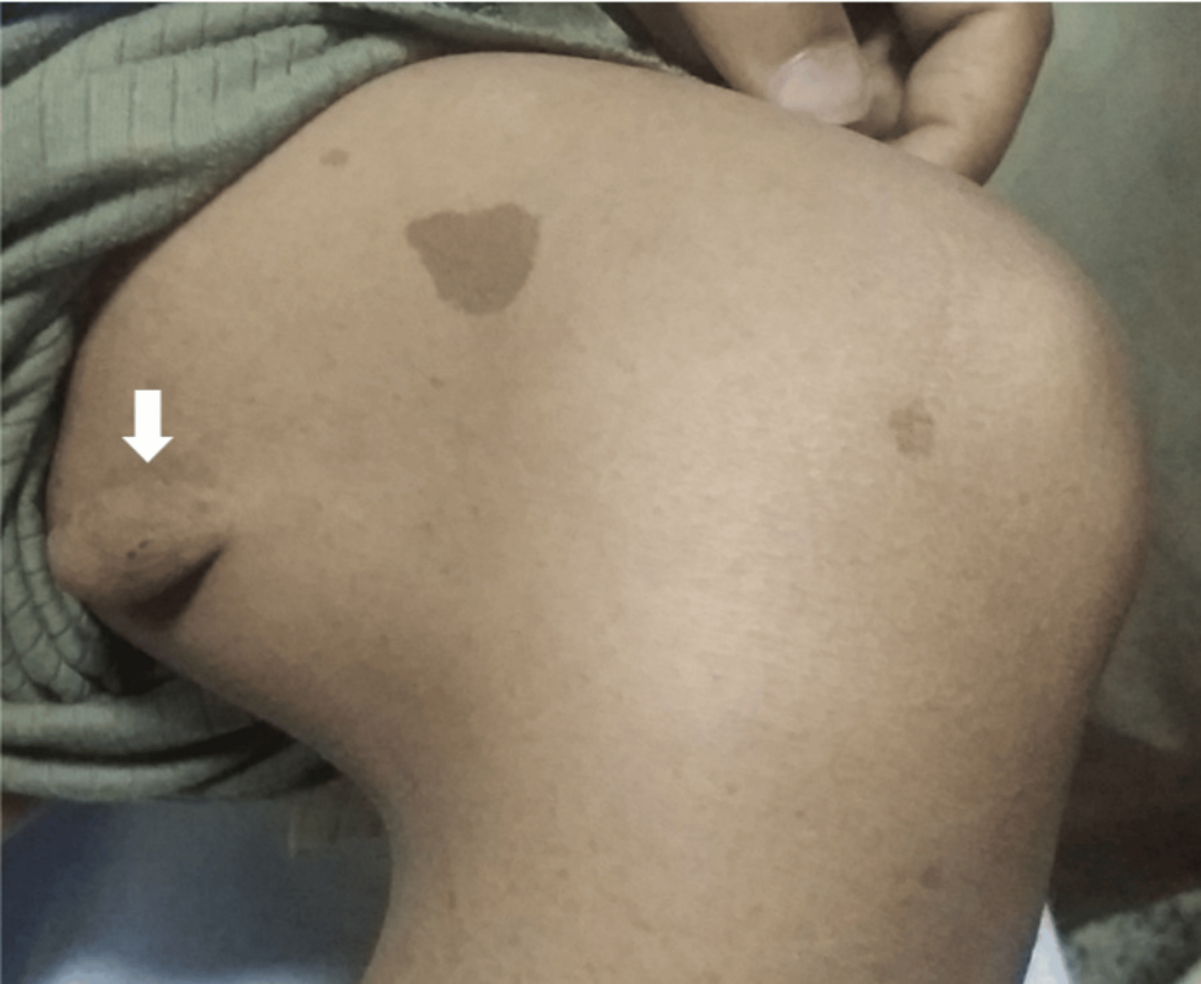
A large, firm oval swelling (arrow), related to the medial aspect of the left lower thigh, with subcutaneous attachment but not attached to the overlying skin "neurofibroma"

​​​​​On presentation, blood pressure was 80/50 in both arms with a heart rate of 120 beats per minute with small volume and normal character and intact peripheral pulsations, respiratory rate of 25 per minute, temperature 37.2°C, saturation of 83% on room air, and body mass index of 19.9. Head and neck examination was significant for increased jugular venous pressure with systolic expansion of neck veins and multiple areas of skin pigmentation “café au lait” patches (Figure 1). Cardiac examination revealed the apex in place, diffuse with systolic retraction. There were palpable left parasternal, epigastric, and pulmonary pulsations with palpable S2. Auscultation was significant for normal S1, accentuated pulmonary component of S2. Abdominal examination was significant for hepatomegaly, multiple café au lait macules (Figure 2), and multiple variable-sized subcutaneous swellings, “neurofibromas” (Figure 3). Examination of the extremities was significant for multiple small areas of skin pigmentations, “freckling” of the axilla. There were multiple skin swellings; the most prominent was a large, firm oval swelling, related to the medial aspect of the left lower thigh, with subcutaneous attachment but not attached to the overlying skin with no overlying color changes (Figure 4). She was noted to have kyphosis of the back. Otherwise, neurological examination was within normal. Fundus examination was within normal.

**Figure 5:**
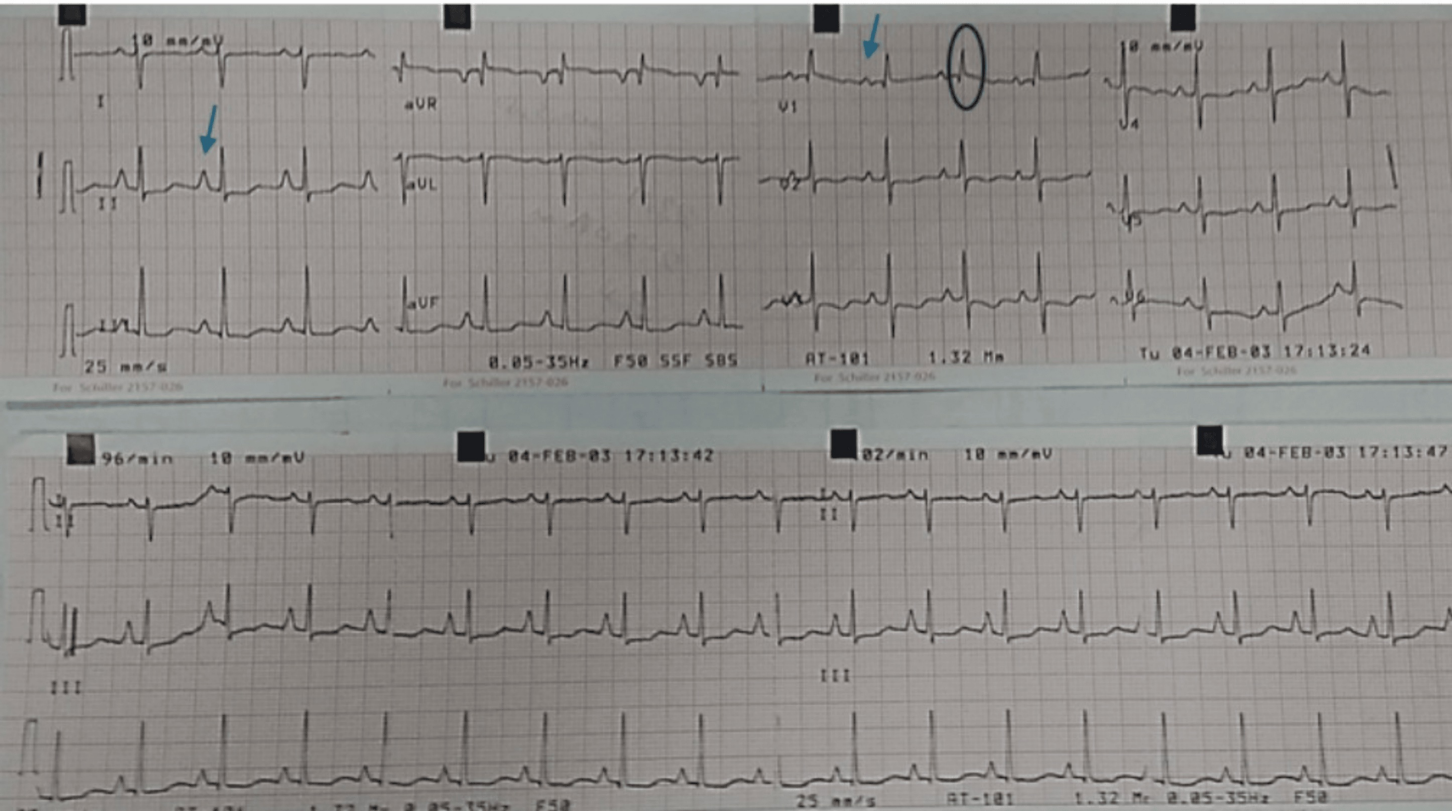
EKG showed sinus rhythm, right axis deviation, with arrows indicate voltage criteria for P-pulmonale and circle around QRS complex in V1 indicates voltage criteria for right ventricular hypertrophy with secondary T wave changes

**Figure 6:**
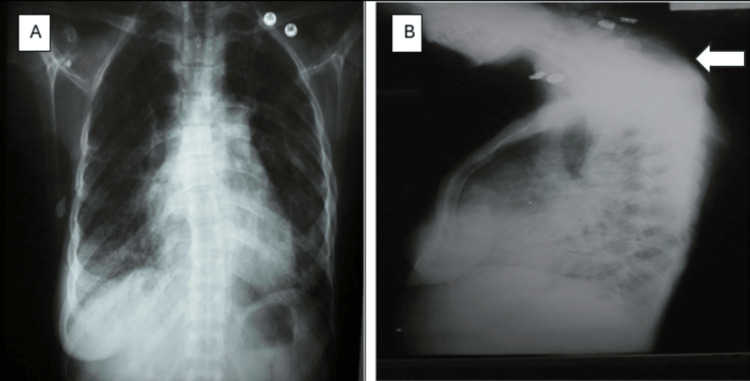
(A) Chest X ray PA view showed cardiomegaly and hyperinflated chest with no evidence of lung congestion or pleural effusion, and (B) chest X ray lateral view, arrow indicates kyphosis PA: posteroanterior.

**Figure 7:**
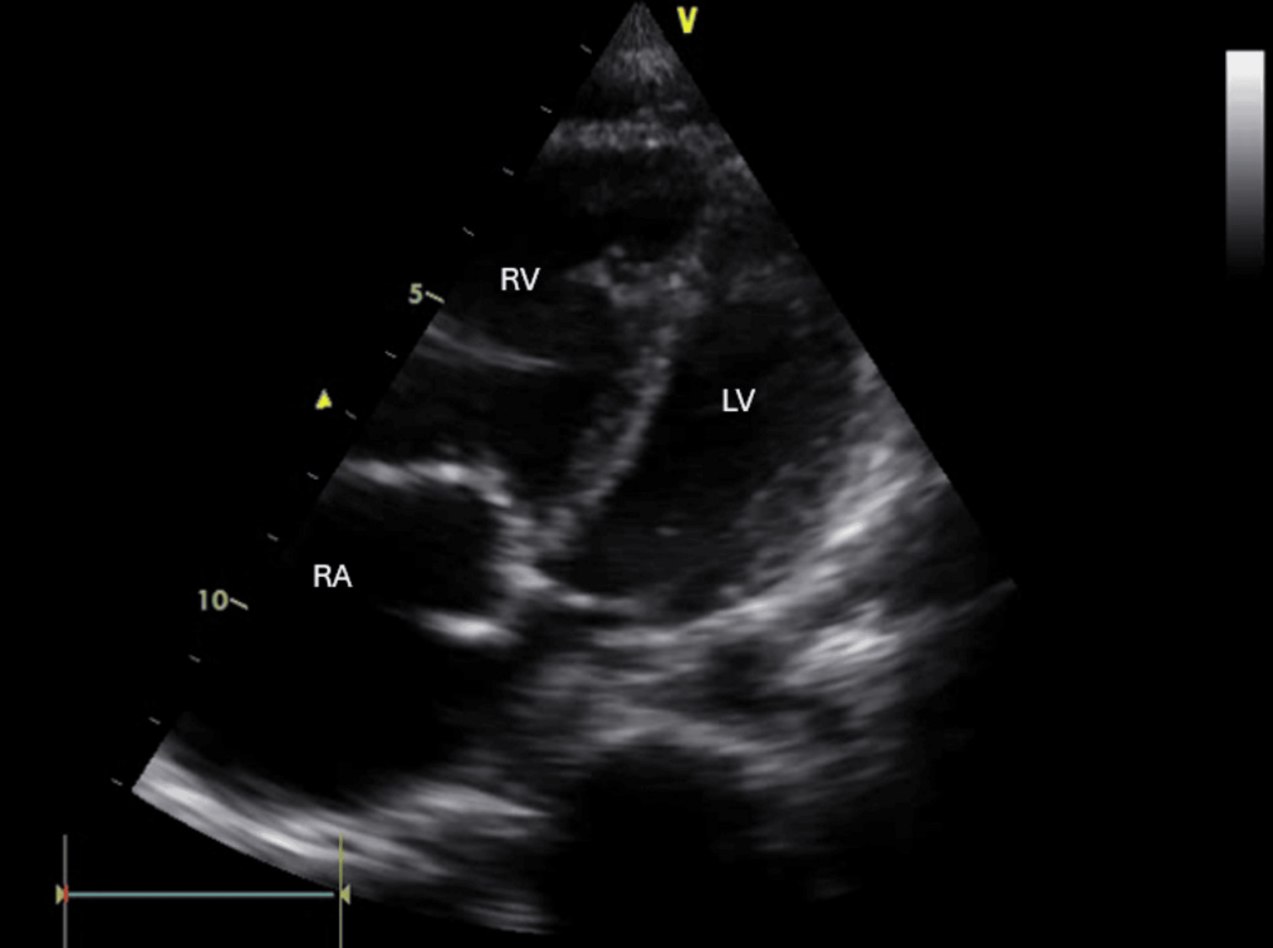
Echocardiography image with apical four chambers view shows normal LV dimensions and significantly dilated RV LV: left ventricle, RA: right atrium, RV: right ventricle.

**Figure 8:**
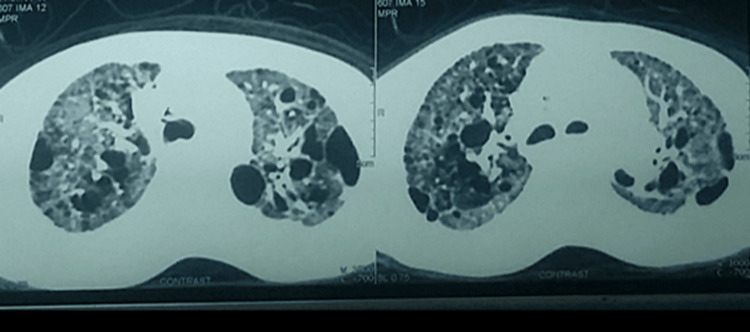
CT chest transverse section shows bilateral diffuse variable-sized lung cysts

EKG was sinus rhythm with voltage criteria for P-pulmonale, right axis deviation, voltage criteria for right ventricular hypertrophy with secondary T-wave changes (Figure 5). Initial laboratory tests were significant for elevated liver enzymes and hypoxemia. D-dimer was positive (Table 1). Chest X ray (CXR) was significant for kyphosis, cardiomegaly, and hyperinflated chest with no evidence of lung congestion or pleural effusion (Figure 6). Transthoracic echocardiogram revealed normal LV dimension and function (EF, 70%), dilated right ventricle with tricuspid annular plane systolic excursion (TAPSE 1.7), severe tricuspid regurgitation, with severe pulmonary hypertension (estimated pulmonary artery systolic pressure (EPASP), 100 mmHg), and dilated non-collapsible inferior vena cava (IVC) (Figure 7). Bilateral lower limb venous duplex showed no evidence of deep venous thrombosis (DVT). CT pulmonary angiography revealed markedly enlarged central pulmonary arteries with pruning of peripheral arteries (suggestive of pulmonary hypertension), with no evidence of pulmonary embolism, enlarged right side of the heart with reflux filling of IVC (suggestive of right-sided cardiac dysfunction), and bilateral diffuse variable-sized lung cysts (Figure 8). Right heart catheterization (RHC), the gold standard for diagnosis, was discussed; however, it was deferred initially due to hemodynamic instability, severe hypoxemia, and clear evidence of advanced right-sided pressures on imaging and echocardiography. Abdominal ultrasound showed congestive hepatomegaly with no evidence of lymphadenopathy or renal masses. Brain and orbital MRI were considered to evaluate for optic pathway glioma and other CNS manifestations; however, given the absence of neurologic symptoms, the study was planned for outpatient follow-up.

**Table 1:** Initial laboratory results PCo_2_: partial pressure of carbon dioxide, HCO_3_: bicarbonate.

Laboratory test	Result	Reference range
International normalized ratio (INR)	1.7	0.8-1.2
Alanine aminotransferase (ALT)	58 U/L	7-56 U/L
Aspartate aminotransferase (AST)	49 U/L	10-40 U/L
Albumin	3.1 g/dL	3.5-5 g/dL
PH	7.46	7.35-7.45
PCo_2_	26 mmHg	35-45 mmHg
HCO_3_	18.5 mmol/L	22-28 mmol/L
Oxygen saturation (SO_2_)	83%	≥95%
D-dimer	Positive	Negative

The patient fulfilled criteria for neurofibromatosis. She also has severe pulmonary hypertension in the setting of associated cystic lesions of the lungs, which is Group V pulmonary hypertension. The plan of management was patient education about the importance of avoidance of cigarette smoking and passive smoking, counselling before planned pregnancy, regular vaccination, particularly flu and pneumococcal vaccines, and avoiding air travel. The patient was referred to pulmonary and cardiac rehabilitation services. She was discharged on home oxygen (4 L/min) and torsemide 5 mg tablets once daily. The patient was seen on outpatient follow-up and showed a stable course and improvement of symptoms.

## Discussion

This case highlights a rare but serious vascular complication of neurofibromatosis type 1: pulmonary hypertension. While NF1 is primarily known for its cutaneous and neurologic manifestations, its association with pulmonary vascular disease is underrecognized and often presents with diagnostic and therapeutic challenges. NF1 is caused by mutations of the *NF1 *gene, identified in 1990, which is located at chromosome 17q11.2 [4]. Diagnosis is clinical and based on criteria established by the National Institutes of Health (NIH) Consensus Development Conference, requiring the presence of at least two out of seven specific features. These include multiple café-au-lait macules, neurofibromas, axillary or inguinal freckling, optic gliomas, Lisch nodules, osseous lesions, or a first-degree relative with NF1 [5,6]. In the present case, the patient met the diagnostic criteria for NF1.

Pulmonary hypertension (PH) is a rare but increasingly recognized vascular complication of NF1. A literature review identified 18 reports encompassing 31 cases of pre-capillary PH in patients with NF1, confirmed via right heart catheterization by a mean pulmonary artery pressure (mPAP) ≥25 mmHg and a pulmonary artery wedge pressure <15 mmHg [3]. PH associated with NF1 (PH-NF1) is categorized under Group 5 PH by current clinical guidelines, encompassing cases with unclear or multifactorial mechanisms [6]. Proposed mechanisms include parenchymal lung destruction, chest wall deformities leading to restrictive lung physiology, left heart disease, and primary pulmonary vascular remodeling. Although interstitial lung disease is commonly associated with NF1, one-third of PH-NF1 cases without significant parenchymal involvement suggest a primary vascular pathology [7]. Moreover, the severity of PH in some cases appears disproportionate to the degree of lung involvement, further supporting this hypothesis [3,8].

Clinical characteristics of PH-NF1 include female predominance, late diagnosis, and poor prognosis. Imaging often reveals cystic or bullous changes predominantly in the upper lobes, ground-glass opacities, and reticular patterns, although up to one-third of patients may lack parenchymal disease [8]. Although NF-1 is congenital, pulmonary fibrosis and neurofibromatosis-associated diffuse lung disease (NF-DLD) are traditionally not evident before the patient reaches adulthood, typically appearing in the third or fourth decade of life [8].

Although pulmonary vascular involvement has been documented in NF1, NF1-related vasculopathy typically involves systemic vessels such as the aorta and renal, mesenteric, or coronary arteries. Vascular involvement occasionally takes the form of multiple aneurysms, but more commonly presents as occlusive disease of the smaller arteries [9]. Histopathological studies in patients with NF1-associated pulmonary hypertension (PH) have demonstrated plexiform lesions, concentric intimal fibrosis, and hypertrophy of the pulmonary arteries and veins, findings similar to those observed in pulmonary arterial hypertension linked to connective tissue diseases [10,11]. These vascular changes suggest that vasculopathy is a significant contributor to PH in NF1, carrying a poor prognosis. However, the complexity of these lesions indicates that the pathogenic process may involve multiple overlapping mechanisms rather than a singular vascular insult.

The pathogenesis of NF1-related vasculopathy is not fully understood. Neurofibromin, the protein encoded by the *NF1 *gene, functions as a tumor suppressor and regulator of cellular proliferation through its role as a GTPase-activating protein. Loss of neurofibromin leads to constitutive activation of Ras and downstream signaling pathways such as the mitogen-activated protein kinase (MAPK) and phosphatidylinositol 3-kinase (PI3K)/protein kinase B (AKT)/mechanistic target of rapamycin (mTOR) cascades, contributing to abnormal vascular cell growth [6,7,12]. This dysregulation likely plays a central role in the vascular remodeling observed in NF1-associated PH. In addition, basic studies have shown that NF1+/− mast cells secrete transforming growth factor β (TGF-β), stimulating fibroblasts to increase collagen and extracellular matrix production. TGF-β signaling, which plays crucial roles in PAH, may be aberrantly activated in NF1, contributing to NF1-PH [13]. Moreover, although NF1 is reported to affect men and women with equal frequency, there is a clear predominance observed of reported cases of PH secondary to NF1 in women, thus supporting a possible role for estrogen metabolism [14].

Early detection of pulmonary arterial hypertension (PAH) is critical for improving patient outcomes. Nevertheless, routine screening with echocardiography or pulmonary function tests in individuals with neurofibromatosis type 1 (NF1) is not recommended, given the low prevalence of PAH or diffuse lung disease in this population (<1%). However, diagnostic evaluation for PAH should be promptly initiated in NF1 patients presenting with suggestive symptoms such as unexplained dyspnea, syncope, or fatigue [11].

In a retrospective, population-based study, Jutant et al. identified 49 patients with PH associated with NF1 from a French cohort, the largest series to date characterizing this condition [6]. The study detailed the clinical, radiologic, histologic, functional, and hemodynamic profiles of these patients. Findings confirmed that PH-NF1 typically presents later in the disease course, predominantly affects females, and is almost invariably accompanied by parenchymal lung abnormalities on high-resolution computed tomography (HRCT), most commonly lung cysts and ground-glass opacities. At the time of diagnosis, pulmonary hypertension was generally severe, demonstrated limited responsiveness to PAH-specific therapies, and was associated with a poor prognosis. As a result, early consideration of double-lung transplantation may be warranted in eligible patients [6].

Therapeutic strategies for PH-NF1 remain limited. Despite the initiation of PAH-targeted therapies, such as endothelin receptor antagonists, phosphodiesterase-5 inhibitors, and prostacyclin analogues, clinical outcomes have been suboptimal. Reported benefits include modest improvement in functional class and hemodynamics, although many patients experience worsening hypoxemia and limited gains in exercise capacity [6]. A subset of patients has shown temporary symptom relief with a combination of triple therapy phosphodiesterase-5 inhibitor (PDE5I), endothelin receptor antagonist (ERA), and parenteral proteinoids. Additionally, isolated cases suggest potential benefit from interventions such as atrial septostomy or tyrosine kinase inhibitors like sorafenib [15].

Nonetheless, overall prognosis remains poor. In one series, most patients experienced disease progression despite aggressive therapy, with high rates of death or consideration for lung transplantation [14]. These findings highlight the importance of early referral for transplant evaluation especially in patients with refractory hypoxemia, progressive right ventricular failure, or lack of response to available therapies. While emerging therapies targeting Ras/MAPK and other downstream pathways show promise in preclinical models, clinical trials are needed to assess their efficacy in this population [3].

## Conclusions

This case underscores the rare but clinically significant association between neurofibromatosis type 1 (NF1) and pulmonary hypertension (PH), a complication with high morbidity and limited therapeutic options. It contributes to the growing recognition of PH-NF1 as a distinct clinical entity characterized by a multifactorial pathophysiology and poor prognosis. Given the progressive nature of the disease and modest responses to PAH-targeted therapies, early diagnosis, multidisciplinary management, and timely referral for lung transplantation evaluation are essential. Increased awareness of this complication may aid in earlier detection and prompt intervention, ultimately improving outcomes in this vulnerable patient population.
